# New insights in thyroid diagnosis and treatment

**DOI:** 10.1007/s11154-023-09859-5

**Published:** 2023-12-02

**Authors:** Fabian Pitoia, Pierpaolo Trimboli

**Affiliations:** 1 Head Thyroid Section, Division of Endocrinology, Hospital de Cl?nicas, University of Buenos Aires, Viamonte, Argentina; 2 Servizio di Endocrinologia e Diabetologia, Ospedale Regionale di Lugano, Ente Ospedaliero Cantonale (EOC), Lugano, 6900 Switzerland; 3 Facoltà di Scienze Biomediche, Università della Svizzera Italiana (USI), Lugano, 6900 Switzerland

**Keywords:** Thyroid, Cancer, Microbiota, Graves, Insights

## Abstract

The prevalence of thyroid disease continues to rise. As a consequence, the research in the thyroid field has significantly increased over time. Thus, clinicians, and endocrinologists first, have to be aware of the important continuous progress achieved, in particular of thyroid cancer, to better manage their patients. This themed issue, titled “New Insights in Thyroid Diagnosis and Treatment,” delves deep into contemporary hot topics in thyroid field. These papers included in the present issue are focused on several aspects in this area, such as imaging, molecular analysis, machine learning and radiomics, nuclear medicine, clinical, and laboratory. Seven papers centers around thyroid cancer. Three papers review imaging modalities for thyroid nodule/cancer assessment. Two papers report a comprehensive review of metabolic issues involving thyroid gland. Finally, a large overview about genetics of Graves’ disease is reported in another study. Clinicians will find this issue very interesting.

The global prevalence of thyroid disease is alarmingly high and continues to rise, imposing a substantial burden on healthcare professionals. Among thyroid diseases, thyroid nodules (TNs) represent the most frequent with an estimated prevalence reaching approximately 60%, as ascertained through high-resolution thyroid ultrasound [1]. However, it is noteworthy that a limited proportion of patients diagnosed with thyroid diseases experience oncological or clinically severe thyroid disorders. For instance, only a minority of thyroid nodules (TNs), approximately 5%, ultimately will be malignant [1, 2]. Although there are a number of studies suggesting that there might be environmental risk factors contributing to a larger diagnosis of thyroid cancer, most of the diagnosis can be attributable to the widespread use of ultrasonography, the generalized imaging screening of the thyroid gland, and the higher access to health care [2–5]. According to the National Cancer Institute from the United States of America, the incidence of these tumors has tripled over the last decades, with a low and stable mortality rate. Indeed, more than 60% of its incidence has been attributed to tumors smaller than 1 cm [6]. With this premises, clinicians, and endocrinologists first, have to be aware of significant continuous progress in the field of thyroid disease and in particular of thyroid cancer.

This themed issue, titled “New Insights in Thyroid Diagnosis and Treatment,“ delves deep into contemporary hot topics in thyroid field encompassing imaging, molecular, machine learning and radiomics, nuclear medicine, clinical, and laboratory aspects.

The central theme of seven papers featured in this issue is centered on thyroid cancer. Specifically, Smulever et al. delve into the contemporary subject of active surveillance (AS) of small papillary thyroid carcinoma (PTC). Several international institutions have been launched their protocols, despite ongoing debates surrounding the subject and the necessity for additional prospective evidence. A wealth of global experience underscores a notable trend among these tumors, characterized by minimal changes in size during the active surveillance period. These growth patterns frequently lean towards slow progression or even size reduction. The authors of this review propose a systematic framework for implementing active surveillance, underscoring the pivotal role of meticulous patient selection. A comprehensive update on the current state of molecular testing for thyroid nodules is reported by Ferraz C. Biopsy of TNs often faces the challenge of indeterminate samples (e.g., Bethesda III-IV). While, historically, these cases often led to surgical interventions with only a 20–30% of malignancy postoperatively, molecular tests have emerged as a beacon of hope. This review provides endocrinologists with a usable guide in this field.

Major guidelines underscore the significance of post-surgery staging to assess the risk of disease persistence, recurrence, and mortality. The management of differentiated thyroid cancer (DTC) assessed with the initial risk of recurrence and the dynamic risk assessment to define additional treatments and the understanding of the meaning of the different responses to treatment and their impact in the long-term follow up is clearly discussed by Jerkovich et al. Additionally, in this special issue we find a thorough analysis of those patients with intermediate risk of recurrence. This topic is reported in a comprehensive overview by Padovani et al. with a particular focus on the nuanced criteria that should guide decisions regarding adjuvant therapy in the current era of personalized medicine. As result of that review, unlike their low- and high-risk counterparts, the literature available for intermediate-risk DTC is fraught with contradictions, and a consensus on adjuvant therapy remains elusive. Another paper from authors representing the Latin American Thyroid Society present the challenges for the management of radioiodine (RAI) refractory DTC in Latin America. Identifying RAI-refractoriness is often straightforward in specialized centers, but the timing for starting multikinase inhibitors (MKI), the availability of genomic testing, and the prescription of MKI and selective kinase inhibitors vary globally in Latin America. Access to MKI remains a hurdle across all Latin American countries, extending to new selective tyrosine kinase inhibitors requiring genomic testing, which is not widely accessible. As precision medicine advances, it reveals significant disparities, and despite efforts to enhance coverage and reimbursement, molecular-based precision medicine remains beyond reach for most of these countries. The authors call for attention in order to take urgent measures to bridge the gap between the current *state-of-the-art* care for RAI-refractory thyroid cancer and the existing situation in Latin America. The landscape of genetic alterations of differentiated thyroid cancer (DTC) in the pediatric population is explored by Alina de Sousa et al. Although an infrequent tumor occurring at this age and, despite its aggressive presentation, pediatric DTC exhibits an exceptional good prognosis when compared to its adult counterpart, being seldomly radioiodine refractory. This manuscript contributes to the elucidation of the global molecular landscape of pediatric thyroid cancer, highlighting prevalent alterations that serve as pivotal oncogenic drivers. Piccardo et al. perform a systematic review of the literature on the prognostic value of pre-ablative thyroglobulin levels (pa-Tg). The results of the retrieved studies provide compelling evidence of paTg’s prognostic value in pediatric DTC. Notably, pa-Tg cutoff values were identified as valuable markers. These findings are very useful for clinical practice. Both thyroid autoimmunity and thyroid malignancy are common clinical conditions, and there are clues that a common mechanism exists. This topic is investigated in another review by Valsecchi et al. This manuscript raises important insights that can help physicians to better personalize DTC patient’s clinical management. Additionally, Cunha Leite et al. helps us to understand the concomitant scenario of thyroid autoimmunity and thyroid cancer. Both conditions exhibit shared molecular signatures involving the programmed cell death protein 1 (PD-1)/ programmed cell death ligand 1 (PD-L1) axis, suggesting a common underlying mechanism, and further investigation is warranted to elucidate the molecular link between these conditions for improved patient management.

Three papers included in the present issue are focused on imaging modalities for thyroid nodule/cancer assessment. One comprehensive narrative review by Bojunga et al. provides an overview of thyroid ultrasound (US) and its adjunctive techniques for the evaluation of thyroid nodules. The most relevant data about elastography, superb microvascular imaging, contrast-enhanced ultrasound, and multiparametric ultrasound, is summarized to expand the diagnostic spectrum and enrich the diagnostic toolkit. In addition, it is debated the potential role of artificial intelligence. An overview about machine learning and radiomics in nuclear medicine is also presented by Dondi et al. The fusion of these innovative technologies with nuclear medicine modalities has begun to unlock previously uncharted diagnostic possibilities. This systematic review unveiled a trove of seventeen studies where radiomics and ML flexed their diagnostic muscles across a spectrum of thyroid disease scenarios. From the assessment of thyroid incidentalomas at ^18^F-FDG PET to the evaluation of cytologically indeterminate thyroid nodules and the classification of various thyroid diseases, these technologies showcased their versatility and potential. Imperiale et al. perform a comprehensive summary of most advanced molecular imaging modalities for the management of medullary thyroid carcinoma is included in this issue. This paper can guide endocrinologists and other thyroidologists towards a rationale for the use of these procedures also including theragnostic opportunities to personalize treatment.

Two papers report comprehensive review of metabolic issues involving thyroid. The matter of relationship between thyroid cancer and insulin is intriguing. A narrative review by Brenta et al. is focused on the topic of insulin resistance as the potential mediator of incidence and progression of differentiated thyroid carcinoma. A unanimous suggestion emerged from this paper – there exists a positive association with thyroid cancer. Similarly, in the realm of diabetes, support for a link with thyroid cancer was evident in four out of five publications. A nuanced narrative unfolded in the seven studies probing antidiabetic agents, with a noteworthy indication that metformin might hold promise in benefitting thyroid cancer outcomes. In another paper, the implication of microbiota in the thyroid field in fully explored by Virili et al. This systematic review of published reviews, summarizes the conclusion of 38 reviews, and then achieves very high interest. The conclusions are relevant for clinicians and advice for further studies in this emerging topic.

One large overview about genetics of Graves’ disease is reported in another paper by Grixti et al. Numerous genetic studies were reviewed and discoveries over years were outlined, starting with historic candidate gene studies and then exploring more recent ones. In addition, emerging evidences achieved were discussed. Clinicians have to be aware of these aspects to be capable to implement future targeted clinical therapies of Graves’.

In conclusion, the contents of the current REMD issue stand as a valuable resource for clinicians and clinical investigators specializing in thyroid disorders. The diverse array of papers presented not only deepens our understanding of critical aspects within this field but also serves as a catalyst for further exploration and advancements in thyroid disorder research. The collective insights offered in this issue contribute significantly to the ongoing dialogue, fostering a rich foundation for informed clinical practice and future investigative pursuits.

## Data Availability

No datasets were generated or analysed during the current study.
